# Oxidative Stress and Reproductive Function in the Aging Male

**DOI:** 10.3390/biology9090282

**Published:** 2020-09-11

**Authors:** Paulina Nguyen-Powanda, Bernard Robaire

**Affiliations:** 1 Department of Pharmacology and Therapeutics, McGill University, Montreal, QC H3G 1Y6, Canada; paulina.nguyen-powada@mail.mcgill.ca; 2 Department of Obstetrics and Gynecology, McGill University, Montreal, QC H4A 3J1, Canada

**Keywords:** sperm, advanced paternal age, aging, male reproduction, oxidative stress, reactive oxygen species, DNA damage, antioxidants, male infertility, progeny outcome

## Abstract

With the delay of parenthood becoming more common, the age at which men father children is on the rise. While the effects of advanced maternal age have been well documented, only recently have studies started to focus on the impact of advanced paternal age (APA) in the context of male reproduction. As men age, the antioxidant defense system gradually becomes less efficient and elevated levels of reactive oxygen species (ROS) accumulate in spermatozoa; this can impair their functional and structural integrity. In this review, we present an overview of how oxidative stress is implicated in male reproductive aging by providing a summary of the sources and roles of ROS, the theories of aging, and the current animal and human studies that demonstrate the impacts of APA on the male germ line, the health of progeny and fertility, and how treatment with antioxidants may reverse these effects.

## 1. Introduction

The delay of parenthood is becoming more common due in part to societal pressure of focusing on one’s career, to the availability of effective methods of contraception, and to assisted reproduction technologies. In the United States, the proportion of men over the age of 40 fathering live births has steadily increased from 6.1% (1972–1975) to 12.7% (2011–2015) [1]. While it is well understood that women have a so-called “biological clock”, male reproduction in the context of aging has not been as extensively studied. Since men continuously produce sperm throughout their entire life, starting at the onset of puberty, this in part has instilled the notion that advanced paternal age (APA) is not a crucial factor to consider in the context of reproduction and fertility. Although it is well recognized that advanced maternal age is associated with a wide variety of reproductive complications, such as preterm births, still birth, and infant mortality [2], it is only in the last few years that a number of studies have shown that the impact of APA has important consequences on both fertility and on progeny outcome [3,4].

Male aging is negatively correlated with sperm parameters, reproductive hormone levels, testicular function, chromosomal structure, and sperm DNA integrity, all of which contribute to infertility and detrimental effects on the offspring [5]. In this review, we will provide a summary of current theories of aging and then focus on what is currently known about oxidative stress in the context of reproductive function in the aging male. We will examine how these factors affect the male germline and the health of the progeny since damage from oxidative stress is considered one of the main factors that impairs the functional and structural integrity of sperm in aging men [5,6].

## 2. Theories of Cellular Aging

Aging is defined by a decline in cellular and physiological function in a time-dependent manner [7]. Hallmarks of aging include changes in blood pressure, stride intervals, respiratory cycles, and vision, as well as fertility [8]. There are many theories that have been proposed to explain why and how aging occurs. Some of these include the telomere theory, the immunologic theory, and the free radical theory of aging. These theories have all been proposed to understand the complexity of the aging process and are not mutually exclusive. 

### 2.1. Telomere Theory 

Telomeres, first described by Mclintock and Muller in the late 1930s and early 1940s, are DNA-protein structures of repeating nucleotides (TTAGGG) that are found at the end of chromosomes [9]. They protect chromosomes from nucleolytic degradation and unnecessary recombination and repair. They do so by preventing chromosomes from being detected as double strand breaks [9]. The telomere theory of aging suggests that the shortening of telomeres is directly correlated with aging. According to Hayflick, there is a limit that cells reach before they stop dividing, which is caused by the gradual shortening of telomeres that occurs with each replication during cellular division [10]. This can lead to cellular senescence, apoptosis, or even transformation of somatic cells into oncogenic cells [9]. As telomeres shorten with age, the length of telomeres may be used to predict an individual’s health, life expectancy, as well as how fast the aging process occurs. 

Age-related oxidative stress can also impair the integrity of telomeres by altering telomere length and generating 8-hydroxy-2′-deoxyguanosine (8-oxodG) lesions due to its guanine-rich nucleotide sequence [6,11]. This damage can be quite detrimental and can disrupt the stability of telomeres and the interactions between telomeres [11]. Although advanced aging decreases telomere length in somatic cells, changes in telomere length in germ cells varies depending on the species examined [11]. While studies in mice have shown a decrease in sperm telomere length, human studies indicate that sperm telomere length increases with age [12].

### 2.2. Immunologic Theory 

The immunological theory is also known as the autoimmune theory of aging. This theory was first proposed in 1969 by Walford and states that the immune system declines with age; this can lead to autoimmunity and an increased vulnerability to infections and diseases [13,14]. This is due to the decrease in T-cell function and responses that occurs with age. In addition, errors that occur during DNA replication in immune cells can increase the production of autoantibodies. These antibodies cannot differentiate between the body’s proteins and a foreign antigen and can therefore mount an attack on the normal tissues of the body, leading to autoimmune disease [14].

The blood–testis barrier (BTB) protects post-meiotic germ cells in the seminiferous tubules from harmful substances in the systemic circulation and from immune cells that can mount an attack against spermatids, as these express proteins that are not viewed as self by the immune system [15]. Levy et al. demonstrated that aging impairs the integrity of the BTB in the Brown Norway (BN) rat due to the absence of tight junctions between Sertoli cells [16]. This suggests that aging and a defective BTB may promote the infiltration of immune cells into the testicular microenvironment, leading to degeneration of male germ cells with age [16]. 

### 2.3. Free Radical Theory 

This theory was first introduced in the 1950s by Gerschman and was further described by Harman [17,18]. It states that aging and the age-associated decline in cellular function are due to an accumulation of damage in DNA, protein, and lipid caused by free radicals [10,19]. Reactive oxygen species (ROS) such as superoxide (O2^•−^), hydroxyl (OH^•−^), nitric oxide (NO^•^), and peroxynitrite (ONOO^−^) are highly reactive and unstable free radicals produced as a byproduct of cellular metabolism. These ROS, if not neutralized by antioxidants, can cause deleterious effects of macromolecules in cells and eventually lead to death. Aged tissues have been found to display increased levels of ROS, suggesting either a decrease in antioxidant defense or an increase in ROS production, or both, with age [20]. Today, this theory remains as one of the most widely accepted theories of aging.

## 3. Oxidative Stress

Oxidative stress occurs as a result of an imbalance in the levels of ROS and the antioxidants required for their neutralization. Although some ROS are free radicals, such as superoxide and hydroxyl, ROS also exist in non-radical forms such as hydrogen peroxide (H_2_O_2_). At low endogenous levels, ROS are required to carry out important roles in various physiological functions [21]. However, when levels of ROS reach a point higher than that required physiologically, a condition of oxidative stress occurs that can damage cellular constituents. Oxidative stress is not only associated with aging but is also correlated with diseases such as Alzheimer’s disease, chronic obstructive pulmonary disease, diabetes, and cardiovascular disease [22]. In addition to aging, testicular oxidative stress can also occur due to other factors such as chemotherapy, inflammation, cryptorchidism, varicocele, and exposure to toxic environmental pollutants [23,24,25,26]. 

### 3.1. Source of Reactive Oxygen Species 

The generation of ROS in cells can be due to either endogenous or exogenous factors. Endogenous production of ROS occurs in various organelles that consume oxygen in large amounts, such as mitochondria, peroxisomes, and the endoplasmic reticulum (ER) [27]. In the testes, where steroidogenesis and spermatogenesis occur, the rate of ROS production is very high due to the large consumption of oxygen required for steroid and sperm production [28]. The most common source of endogenous ROS generation, namely superoxide, occurs through leakage from the electron transport chain in the mitochondria during cellular respiration. Enzymes in the electron transport chain such as nicotinamide adenine dinucleotide phosphate (NADPH) oxidase and cytochrome p450s are responsible for generating various different ROS in the mitochondria [27,28]—for instance, the formation of NO performed by NO synthase in the presence of NADPH via the oxidation of L-arginine to L-citrulline [29]. In peroxisomes, hydrogen peroxide is mainly produced via the transfer of electrons to oxygen from a variety of metabolites [27]. In the ER, enzymes such as cytochrome p450 and b5 and diamine oxidase are responsible for the endogenous generation of ROS [30]. Exposure to external sources can also trigger an increase in ROS production in the cells. Some exogenous sources include air and water pollution, alcohol, tobacco smoke, pesticides, ultraviolet light, and various drugs [27].

### 3.2. Molecular Effects/Function of ROS 

ROS are often discussed in the context of cellular damage and their detrimental effects on health. However, ROS have dual roles and can also act as signaling molecules in various physiological functions. ROS are involved in many signaling pathways such as cytokine, insulin, growth factor, AP-1, and NF-κB signaling [30]. With respect to the male reproductive system, low levels of ROS are required for normal sperm function and thus fertilization, whereas elevated levels are toxic and may lead to infertility due to oxidative damage [31]. ROS are involved in a wide variety of functions ranging from the regulation of spermatogenesis to capacitation and the acrosome reaction. Many of the processes carried out by ROS in spermatozoa are dependent on its activation of tyrosine phosphorylation. For instance, H_2_O_2_ is involved in sperm capacitation by activating tyrosine phosphorylation, and thus the cell signaling cascade [31]. In addition, it has been shown that antioxidants inhibit the acrosome reaction, which requires abundant levels of ROS, highlighting the need for ROS in sperm [31]. The motility of the sperm flagella is also dependent on ROS since it is used to induce hyperactivation. This process requires tyrosine phosphorylation, which is facilitated by ROS [31].

### 3.3. Metabolism and Reproductive Function of Nitric Oxide

NO is primarily metabolized into nitrite and nitrate, which can be found in various biological fluids [32,33]. The metabolism of NO is required in the body since it is involved in many physiological systems, such as the nervous system, the circulatory system, the immune system and the reproductive system [34,35,36,37,38,39]. In males, NO synthases are found in both human and rat testes and epididymides, suggesting a likely role for NO in spermatogenesis [40]. Furthermore, it has been shown that NO synthase is involved in the process of fertilization, including sperm motility and the acrosome reaction in mice [41]. At low concentrations, NO stimulates capacitation, zona pellucida binding, and human sperm motility, which are necessary for fertilization and reproduction [42,43,44]. In contrast, seminal plasma concentrations of nitrite, a stable metabolite of NO, are not associated with sperm concentration, sperm motility, leukocytospermia, or sperm culture [45].

### 3.4. Redox Imbalance and Germ Cell Damage

**Figure 1:**
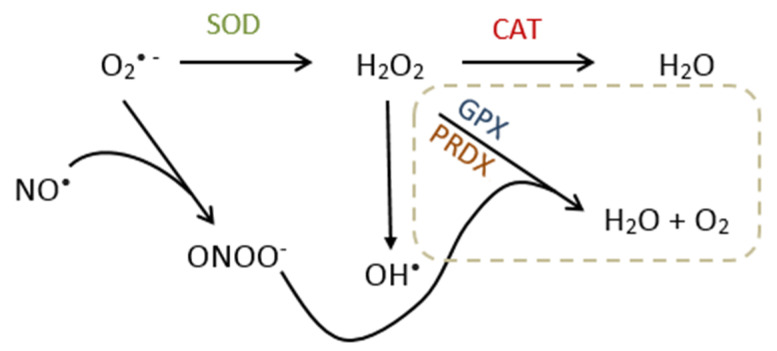
Reactive Oxygen Species and Antioxidant pathway. Superoxide (O_2_^• −^) is broken down by superoxide dismutase (SOD) into hydrogen peroxide (H_2_O_2_), which is further neutralized into water (H_2_O) by catalase (CAT), glutathione peroxidase (GPX), or peroxiredoxins (PRDXs). If not neutralized, H_2_O_2_ can break down into hydroxyl (OH^•^). The reaction between superoxide and nitric oxide (NO^•^) generates peroxynitrite (ONOO^−^), which is scavenged by GPX and PRDX.

ROS that accumulate in the cells must be neutralized by antioxidant enzymes in order to maintain redox balance and to prevent oxidative stress. The breakdown of O_2_^• −^ into H_2_O_2_ is done by an antioxidant enzyme called superoxide dismutase (SOD). All three SOD isoforms, namely cytoplasmic SOD1, mitochondrial SOD2, and extracellular SOD3, are capable of breaking down O_2_^• −^ in H_2_O_2_ [46]. H_2_O_2_ is then further neutralized by catalase (CAT) into water. There are other antioxidant enzymes responsible for the neutralization of H_2_O_2_ and ONOO^−^, including glutathione peroxidase (GPX) and peroxiredoxins (PRDXs), which exist in eight and six isoforms (GPX1-8, PRDX1-6), respectively (Figure 1) [47,48]. Oxidative stress and cellular damage occur when levels of ROS are higher than levels of antioxidants [49].

#### 3.4.1. Lipid Peroxidation 

The plasma membrane of sperm has a large component of unsaturated fatty acids, thus rendering it susceptible to harmful attacks by free radicals and ROS [6]. As a result, lipid peroxidation occurs, which can lead to the formation of electrophilic aldehydes, such as 4-hydroxynonenal (4HNE), acrolein, and malondialdehyde (MDA) [6]. These small molecules are particularly harmful to the cell as they have the potential to trigger the generation of more ROS, which in time makes the sperm functionally defective due to damage in the plasma membrane and unable to fuse with the oocyte for fertilization [6]. 

#### 3.4.2. DNA Damage 

As a consequence of oxidative stress, nucleic acid base damage, such as 8-oxodG lesions, can form in sperm DNA. The attack of ROS on guanine in DNA is easily facilitated due to guanine’s high redox potential. 8-oxodG is commonly used as a marker of oxidative damage in DNA, and it has been shown to be elevated in the sperm of infertile male patients [6]. Terminal deoxynucleotidyl transferase dUTP nick end labeling (TUNEL) assays, which detect DNA fragmentation by labeling the 3′-hydroxyl termini in the double-strand DNA breaks, have demonstrated that 8-oxodG and sperm DNA damage are highly correlated, suggesting that most of the DNA damage that occurs in sperm is due to oxidation [6]. As a consequence, oxidative damage on DNA can lead to inhibition of DNA replication and incorrect DNA repair [50]. DNA damage that is left unrepaired in aged male germ cells can be passed on to the future generation, possibly having negative consequences on the health of the progeny. 

#### 3.4.3. Modifications to Proteins

Approximately 70% of the oxidized molecules in cells are proteins, suggesting that proteins are the main targets of ROS [51]. Modifications to proteins due to oxidative stress can lead to impaired protein functions or to the activation/inactivation of signaling pathways required for normal sperm physiology [51,52]. These modifications include thiol oxidation, 4HNE protein adducts, tyrosine nitration and sulfonation, all of which have substantial effects on reducing the motility of sperm in both humans and rats [52].

## 4. Animal Studies and the Effects of Aging and Oxidative Stress in Male Germ Cells

Various animal models and model organisms exist to describe oxidative stress and the process of aging, including *Caenorhabditis elegans*, *Drosophila melanogaster*, mice, rats, domestic dogs, and non-human primates [53]. These models are used to determine the molecular changes that occur with age and to identify key factors that regulate longevity. Rodents, and particularly mice, are often used as animal models for studying male reproductive aging because the size of the mouse genome is similar to that of humans and a similar number of genes are encoded [53]. In addition, genetic modification in mice is relatively easy to do in order to study aging in the context of oxidative stress, by either knocking out or overexpressing antioxidant genes [54]. According to the National Institute on Aging, BN rats are also a reliable animal model to use in aging studies as they live longer than 36 months without contracting any age-related diseases that have been previously observed in other strains [16,55,56,57,58,59].

### 4.1. Effects of Oxidative Stress in Aged Transgenic Mice

Superoxide dismutase 1 (SOD1) and CAT are enzymatic antioxidants in the ROS neutralization pathway that break down O_2_^• −^ and neutralize H_2_O_2_, respectively. A study done by Selvaratnam and Robaire investigated the effects of aging and oxidative stress in testicular male germ cells in *Sod1* and *Cat* knockout (KO) mice [26]. They found that fertility, Sertoli cells, and germ cells were reduced in all aged mice [60]. However, aged *Sod1^−/−^* mice had a further decrease in fertility compared to their age-matched controls. Furthermore, aging and *Sod1* KO resulted in a higher number of spermatozoa with oxidized DNA (8-oxodG) [60]. When spermatocytes were treated with the pro-oxidant molsidomine (SIN-10), aged *Cat^−/−^* mice were able to neutralize the ROS whereas levels of ROS remained elevated in aged wild-type (WT) and *Sod1^−/−^* mice [60]. This suggests that aged *Cat^−/−^* mice have compensatory mechanisms in place to neutralize ROS, namely an upregulation of peroxiredoxin-1 (*Prdx1*), whereas aged wild-type and *Sod1^−/−^* mice do not [60]. This indicates that SOD1 is a crucial antioxidant enzyme for protecting germ cells from oxidative damage. 

A study performed by Noblanc et al. also demonstrated that in mice with a null mutation for *Sod1,* there is an exacerbation of the effects of aging and oxidative stress in the epididymis, where spermatozoa become mature [61]. Their findings show that aging increased oxidative DNA damage (8-oxodG) and lipid peroxidation (4HNE) in the epididymis, where a further increase was observed in *Sod^−/−^* mice [61].

Another antioxidant enzyme required to maintain the integrity of sperm is PRDX, which neutralizes hydrogen peroxide, organic hydroperoxides, and peroxynitrite (ONOO^−^) [62]. A study done by Ozkosem et al. demonstrated that peroxiredoxin-6 (*Prdx6*) knockout negatively affects sperm function, sperm chromatin quality, and fertility in aged mice [62]. They found that DNA fragmentation and DNA oxidation (8-oxodG) in sperm increased with age with a greater amount of damage in the *Prdx6^−/−^* mice. In addition, aging further exacerbated the fertility issues that were observed in young *Prdx6^−/−^* mice, where litter and pup size are significantly decreased. They postulated that these negative reproductive outcomes were likely due to the decrease in sperm motility and the extensive amount of damage in the paternal DNA, along with high levels of lipid peroxidation [62]. This highlights the importance of PRDX6 in maintaining sperm quality and function by preventing damage caused by age-related oxidative stress. PRDX4 is found in all tissues, including the testes, and is localized in the endoplasmic reticulum [63]. Mouse knockout models of *Prdx4* showed no change in fertility but an increase in germ cell loss, mainly spermatids, leading to testicular atrophy [47,64]. Although studies investigating the role of other PRDX isoforms in aging animals are limited, human studies have revealed that a decrease in PRDX1 and 2 in the seminal plasma is associated with a decrease in sperm concentration and an increase in lipid peroxidation and sperm DNA damage [65]. Similar to PRDX6, PRDX1 and 2 are localized in the cytosol, mitochondria, nuclei, and peroxisomes [47].

PRDX activity depends on thioredoxin/thioredoxin reductase reactivation. If disrupted, or reduced such as in aging males, this leads to a decrease in sperm motility and capacitation and an increase in sperm DNA damage, resulting in male infertility [66,67]. In sperm, specific forms of thioredoxins called thioredoxin domain-containing proteins (Txndc) are expressed [68]. A study by Smith et al. investigated the effects of inactivating *Txndc2* and *Txndc3* in mice. [68]. They found no change in spermatogenesis, epididymal sperm maturation, or fertility in *Txndc2^-/-^*, *Txndc3^-/-^*, and double transgenic mice at the age of 3–9 months [68]. However, sperm motility drastically decreased in an age-dependent manner in the transgenic mice compared to the WT [68]. Furthermore, aged transgenic mice displayed impaired chromatin protamination and higher levels of sperm with increased ROS, lipid peroxidation, and DNA damage [68]. The results obtained from this study demonstrate that thioredoxins also contribute to protecting sperm from age-related oxidative damage. 

The eight isoforms of GPX function in various different locations and cellular compartments, such as the cytosol, mitochondria, intestinal epithelium, and plasma [48]. GPX5 plays an important role in male fertility as it is specific to the epididymis [48]. A study conducted by Chabory et al. investigated the role of epididymal GPX5 in mouse sperm by generating *Gpx5* knockout male mice [69]. They found significantly higher levels of sperm with 8-oxodG lesions in *Gpx5^-/-^* mice compared to the WT control [69]. In addition, aged (14 months) Gpx5^-/-^ mice displayed stronger signals of 8-oxdG in the cauda epididymis compared to WT mice of the same age [69]. When mated with WT female mice, young (3 months) Gpx5^-/-^ male mice exhibited normal fertility and mating behavior, with a similar litter size to WT male mice [69]. However, an increased frequency of miscarriages and developmental defects was observed with age when mating with aged Gpx5^-/-^ male mice over 12 months old [69]. Moreover, a higher number of dead pups and aborted fetuses when matings were done at 13 and 16 months in Gpx5^-/-^ male mice was noted [69]. This suggests that the effects of GPX5 knockout are more pronounced with age and that damaged sperm DNA may be the cause of the negative reproductive outcomes seen in aged Gpx5^-/-^ mice. 

### 4.2. Effects of Oxidative Stress in Aged Brown Norway Rats

Using BN rats to study aging in the male reproductive system, it was shown that aging negatively affects the development of the embryo, fertility, and progeny outcomes [70]; these abnormalities were proposed to be due to age-related hypermethylation of ribosomal DNA in sperm [71]. Genes in the base excision repair pathway are downregulated with age in the pachytene spermatocytes of BN rats, resulting in the increase of 8-oxodG lesions in the germ cells and spermatozoa [72]. This demonstrates that DNA repair mechanisms are also impaired with age. Selvaratnam et al. investigated the response of male germ cells to aging and oxidative stress. They found that aged germ cells have a decrease in cell viability, higher levels of ROS, a greater incidence of apoptosis, and an increase in DNA damage in spermatocytes [73]. Upon treatment of the pro-oxidant, morpholinosydnonimine (SIN-1), young germ cells responded by upregulating the expression of *Sod1*, *Cat,* and *Prdxs*, whereas gene expression of these antioxidants was lower in the germ cells of aged rats. This suggests that germ cells from young and aged rats do not respond to oxidative stress in the same way and that it is more challenging for aged germ cells to maintain redox balance [73].

A study by Weir and Robaire showed that ROS production increases with age in BN rats [74]. In spermatozoa of aged BN rats, superoxide and hydrogen peroxide generation was significantly increased compared to the young WT control [74]. Lipid peroxidation was also significantly greater in the aged BN rats [74]. Furthermore, the enzymatic activity of GPX1, GPX4, and SOD decreased in aged spermatozoa [74]. The results from this study suggest that not only is aging associated with a decrease in the antioxidant defense system but that an increase in ROS production also contributes to the oxidative damage seen with age.

### 4.3. Treatment of Oxidative Stress in Animals 

Several studies have explored how antioxidant treatment or overexpression of antioxidants may be beneficial in the process of aging and in preventing oxidative stress. Studies looking at the overexpression of *Cat* have consistently shown that mice with overexpressed *Cat* have a reduction in age-related pathologies and an increase in lifespan [75]. In contrast, overexpressing *Sod1* was shown to make no difference in increasing lifespan in mice [76]. Dietary supplementation of antioxidants such as vitamin E or vitamin C has caused a reduction in oxidative stress by decreasing lipid peroxidation in the liver of short-tailed field voles; however, no change in oxidative DNA damage was observed in their hepatocytes and lymphocytes [77]. Interestingly, these antioxidants actually shortened lifespan of the voles in both warm and cold conditions, rather than increasing their lifespan as one might expect [77].

There are limited studies looking at how antioxidants may help reverse the effects of aging in male germ cells. However, in the study mentioned previously, Selvaratnam et al. found that pre-treating germ cells with the synthetic antioxidant EUK-134 before SIN-1 treatment decreased levels of ROS in germ cells in young rats only [73]. Nevertheless, EUK-134 did not reverse the decrease in antioxidant expression in spermatocytes of aged rats [73]. Despite having properties of both SOD and CAT, the effects of this synthetic antioxidant as a therapeutic agent have not yet been studied in the context of male reproductive aging.

## 5. Effects of Aging and Oxidative Stress on Human Spermatozoa 

Although there is no fixed age that is used to define APA, it is most commonly characterized as men over the age of 40 years [78]. Current studies focusing on APA involve looking at changes in sperm parameters, sperm DNA, fertility, and progeny outcome [79].

### 5.1. Effects of Aging on Sperm Quality 

Assessment of the changes in sperm parameters (sperm concentration, motility, morphology, and ejaculate volume) is generally used for evaluating fertility in men [5,78]. Despite the latter, the idea that semen quality declines with age remains debatable [78]. Although it is likely that aging and oxidative stress negatively impact sperm quality and thus fertility, there still exists much controversy over whether the assessment of sperm parameters is useful as an indicator of reproductive success or failure [78]. 

When studies controlled for confounding variables (abstinence period, medical history, drug use), a negative relationship between semen volume and age was consistently noted [80,81,82,83,84]. Most studies looking at the changes in sperm morphology showed a general reduction of sperm with normal morphology with advancing age [78]. Nevertheless, some found no relationship between age and sperm morphology [78,85,86]. When considering sperm count and sperm concentration, multiple studies found a decrease in sperm concentration with age (although one study actually showed a 0.3–3.3% increase for each successive year of aging) or no relationship between paternal age and sperm concentration [87,88,89,90]. The challenge in obtaining conclusive data on sperm concentration may be due to the variation in semen volume that occurs with advanced paternal age [88]. Numerous studies have shown a decrease in sperm motility with advanced paternal age; Auger et al. demonstrated a decrease of 0.6% in motility per year of aging [80,81,82,83,84,87,91,92]. However, other studies conducted by Wang et al. and Irvine et al. found that a 0.06% increase in motility occurs with each year of aging [93,94]. Overall, the weight of evidence indicates that sperm motility decreases with age [78].

### 5.2. Effects of Aging and Oxidative Stress on Sperm DNA

As seen previously in animals, aged men also display higher levels of DNA damage, in part due to oxidative stress and the increased number of mitotic divisions during spermatogenesis that occur with age [78]. A study looking at 66 males between the ages of 20–57 years showed that sperm from men over the age of 35 had significantly more DNA breaks than younger men [95]. Furthermore, aging results in impaired sperm chromatin integrity and increased breaks in sperm DNA in infertile men compared to fertile men [96,97]. A study done by Wyrobek et al. investigating 97 men aged between 22–80 years found a significant positive relationship between aging and the percentage of sperm with DNA fragmentation and mutations in FGRF3, a gene associated with achondroplasia [98]. However, aging was not significantly associated with increases in immature sperm chromatin, mutations in FGFR2, an Apert syndrome gene, sperm diploidies, or aneuploidies [98]. Roughly 80% of DNA fragmentation observed in sperm is due to oxidative stress, which, in addition to aging, can arise as a result of frequent infections, obstruction, inflammation, and in some cases, male infertility [5,78]. Rolf et al. conducted a cross-sectional retrospective study demonstrating that infections of accessory sex glands increased with age in infertile men [99], underscoring the complexity of male reproductive aging as there exist many different factors that contribute to aging and oxidative stress. 

### 5.3. Effects of Aging and Oxidative Stress on Fertility and Progeny Outcome

Although data focusing on the impact of APA on fertility are limited, the evidence suggests that the fertility rate decreases with paternal age [78]. An observational study done by Hassan and Killick demonstrated a five-fold increase in time to pregnancy in couples where the male partner was over the age of 45 years [100]. They also found that men over 45 years old were 4.6 times less likely to impregnate their female partners after one year of regular unprotected sex compared to younger men (<25 years old) [100]. In addition, men of APA were 12.5 more likely to be amongst the men who took over 2 years to attain pregnancy in their partners [100]. Based on these data, they concluded that maternal age did not contribute to the decrease in fertility, suggesting that the APA was the main factor [100]. Interestingly, Cocuzza et al. found that fertile men over the age of 40 years exhibited higher levels of ROS in their ejaculate [101]. They rationalize that because elevated ROS is involved in the etiology of male infertility, the chances of inducing pregnancy gradually decrease with increasing paternal age [101].

**Figure 2:**
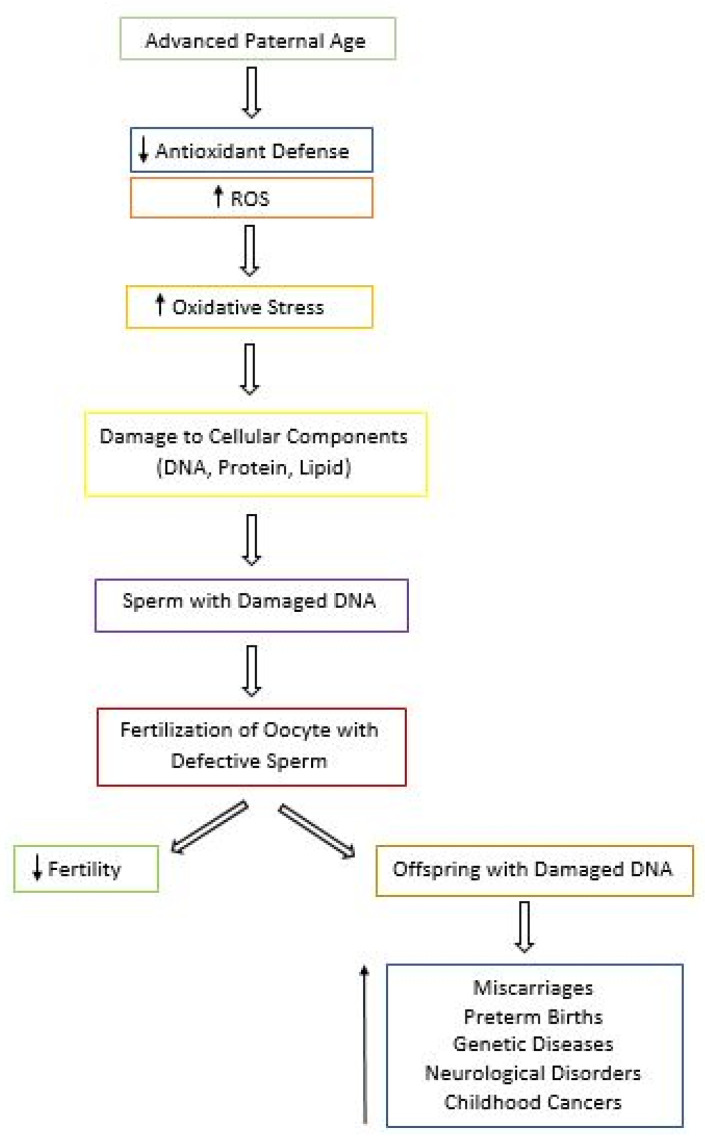
Flowchart of the Consequences of Advanced Paternal Age (APA). With APA, the antioxidant defense system decreases and levels of reactive oxygen species (ROS) increase, leading to oxidative stress and cellular damage. Defective sperm can cause a decrease in fertility. In addition, damage can be passed on to the offspring, resulting in a wide array of consequences for the future generation.

In addition to contributing to male infertility, oxidative stress paired with aging can present other reproductive risks due to the extensive amount of damage seen in aged sperm (Figure 2) [102,103]. APA has been associated with a higher risk of miscarriages, preterm births, late fetal death, and low birth weight [102]. According to the National Birth Defects Prevention Study, after controlling for confounders, APA was found to be linked to increased risk of birth defects in the offspring, such as cleft lip, diaphragmatic hernia, right ventricular outflow tract obstruction, and pulmonary stenosis with each year of aging [103]. In addition, the risk of diseases and genetic disorders including schizophrenia, autism, and bipolar disorder seems to also increase in the offspring of older men [5,104]. Other conditions linked to APA include achondroplasia, Apert syndrome, Klinefelter syndrome, and childhood cancers [5,103]. Thus, in couples where the man is older, genetic counseling should be recommended prior to conception in order to increase awareness of the risk associated with APA [103]. 

### 5.4. Value of Antioxidant Supplementation in Aging Males

There are very few studies looking both at aging and oxidative stress in the context of male reproduction. In addition, many studies investigating the benefits of antioxidant supplementations have limitations. In a recent Cochrane review, it was noted that amongst the 48 randomized controlled trials on dietary oral antioxidant supplementation, most studies were poorly conducted, and therefore no clear conclusion could be drawn regarding the beneficial effects of antioxidants on live birth rates and clinical pregnancy in subfertile men [105]. These limitations include inadequate reporting of the study methods and the adverse effects, small sample size, and low number of trials [105]. In contrast, Greco et al. have described benefits of oral vitamin C and E supplementation in reducing DNA fragmentation in infertile men [106]. In a cross-sectional study by Schmid et al., it was demonstrated that aged men (>44 years) with the highest intake of vitamin C, vitamin E, or zinc had 20% less DNA damage in their sperm when compared to aged men with the lowest intake [107]. Interestingly, the levels of sperm DNA damage in older men taking the highest doses of antioxidants were comparable to those seen in younger men [107]. These data suggest that using dietary supplementation of antioxidant may have the potential to restore the damage seen in the DNA sperm of men of APA, thus possibly improving fertility and progeny outcome. However, another study conducted by Silver et al. did not find any improvements in sperm chromatin integrity with increased intake of vitamin C, vitamin E, or beta-carotene. This demonstrates the lack of consistency between studies investigating the effects of antioxidants on sperm and the need for more well-controlled studies [108]. 

Another antioxidant of interest is coenzyme Q10 (CoQ10), which is found in mitochondria at the midpiece of sperm [109]. Its reduced form, ubiquinol, is an antioxidant that protects sperm plasma membrane from lipid peroxidation [109]. In a prospective study, it was shown that treatment of CoQ10 increases sperm concentration, progressive and total sperm motility, antioxidant capacity, and SOD and CAT activity in men with idiopathic oligoasthenoteratozoospermia (OAT) [110]. At a higher dose, both semen parameters and antioxidant status were further increased, suggesting that CoQ10 may be beneficial in treating idiopathic OAT [110]. However, although a meta-analysis done by Lafuente et al. confirmed that CoQ10 improves sperm parameters, CoQ10 does not increase pregnancy rates in infertile men [111]. Moreover, we are not aware of any data in the literature indicating any improvement of live births in men using CoQ10 [111]. It is therefore necessary to conduct more robust and powered clinical trials in order to find better therapies for male infertility [111]. Table 1 summarizes the effects of antioxidant treatment in aging and infertile men (Table 1). 

## 6. Conclusions

The process of aging and its impact on the male germ line is beginning to be understood. As discussed in the review, there exist many hypotheses exploring how age-related defects occur in the male reproductive system. These include a decrease in the antioxidant defense system and DNA repair machinery and an increase in ROS production, all of which are still not completely defined in the context of paternal aging, male infertility, and reproductive outcome. Furthermore, there is an urgent need for additional studies to obtain a more comprehensive appreciation of how aging and oxidative stress impact sperm chromatin quality. Considering the detrimental effects that have been reported in both animal and human studies, it is also important to be aware of the consequences that may arise when older men plan to conceive children.

## Figures and Tables

**Table 1:** Summary of the Effects of Antioxidant Treatment in Aging and Infertile Men.

Authors/Study	Antioxidant Treatment	Sample	Findings
Cochrane Review [105]	Dietary antioxidant supplementation	Subfertile men	No effect on live birth rates and clinical pregnancy.
Greco et al. [106]	Oral vitamin C and E	Infertile men	Reduced DNA fragmentation.
Schmid et al. [107]	Vitamin C and E, zinc	Aged men (>44 years)	20% less sperm DNA damage.
Silver et al. [108]	Vitamin C and E, beta	Healthy men	No improvement in sperm chromatin integrity and no benefit for fertility issues.
Alahmar et al. [110]	CoQ10	Men with oligoasthenoteratozoospermia (OAT)	Increase in sperm concentration and motility, antioxidant capacity, and SOD and CAT activity.
Lafuente et al. [111]	CoQ10	Infertile men	Improvement in sperm parameters. No change in pregnancy rates. No data on live births.
